# ArthroNavi framework: stereo endoscope-guided instrument localization for arthroscopic minimally invasive surgeries

**DOI:** 10.1117/1.JBO.28.10.106002

**Published:** 2023-10-14

**Authors:** Zhongjie Long, Yongting Chi, Xiaotong Yu, Zhouxiang Jiang, Dejin Yang

**Affiliations:** a Beijing Information Science & Technology University, School of Electromechanical Engineering, Beijing, China; b Guang’anmen Hospital, China Academy of Chinese Medical Sciences, Beijing, China; c Beijing Jishuitan Hospital, Capital Medical School, 4th Clinical College of Peking University, Department of Orthopedics, Beijing, China

**Keywords:** interventional systems, pose estimation, instrument localization, stereo endoscope, electromagnetic tracking, osteochondral autograft transplantation (mosaicplasty).

## Abstract

**Significance:**

As an example of a minimally invasive arthroscopic surgical procedure, arthroscopic osteochondral autograft transplantation (OAT) is a common option for repairing focal cartilage defects in the knee joints. Arthroscopic OAT offers considerable benefits to patients, such as less post-operative pain and shorter hospital stays. However, performing OAT arthroscopically is an extremely demanding task because the osteochondral graft harvester must remain perpendicular to the cartilage surface to avoid differences in angulation.

**Aim:**

We present a practical ArthroNavi framework for instrument pose localization by combining a self-developed stereo endoscopy with electromagnetic computation, which equips surgeons with surgical navigation assistance that eases the operational constraints of arthroscopic OAT surgery.

**Approach:**

A prototype of a stereo endoscope specifically fit for a texture-less scene is introduced extensively. Then, the proposed framework employs the semi-global matching algorithm integrating the matching cubes method for real-time processing of the 3D point cloud. To address issues regarding initialization and occlusion, a displaying method based on patient tracking coordinates is proposed for intra-operative robust navigation. A geometrical constraint method that utilizes the 3D point cloud is used to compute a pose for the instrument. Finally, a hemisphere tabulation method is presented for pose accuracy evaluation.

**Results:**

Experimental results show that our endoscope achieves 3D shape measurement with an accuracy of <730  μm. The mean error of pose localization is 15.4 deg (range of 10.3 deg to 21.3 deg; standard deviation of 3.08 deg) in our ArthroNavi method, which is within the same order of magnitude as that achieved by experienced surgeons using a freehand technique.

**Conclusions:**

The effectiveness of the proposed ArthroNavi has been validated on a phantom femur. The potential contribution of this framework may provide a new computer-aided option for arthroscopic OAT surgery.

## 1 Introduction

### 1.1 Background and Motivation

This study is motivated by the clinical need for surgical navigation in minimally invasive arthroscopic surgery. An example of minimally invasive arthroscopic surgery is osteochondral autograft transplantation (OAT),^1^ which is an option for repairing focal cartilage defects in the knee ioints. OAT is a useful treatment for small osteochondritis dissecans ($<2\;{\mathrm{cm}}^{2}$)^2^^,^^3^ and works by replacing a focal cartilage defect area with one or more osteochondral autografts generally harvested from the non-weight-bearing area of the patient’s healthy cartilage and bone. OAT makes small incisions and thus is usually performed arthroscopically and is minimally invasive. In contrast, mosaicplasty is a surgical technique that involves inserting three or more small plugs of healthy cartilage and bone from a non-weight-bearing site to fill larger areas of cartilage defects, forming a mosaic appearance.^4^^,^^5^ Mosaicplasty is usually performed through open incisions, which is more invasive and requires a longer recovery time. OAT can be conducted by open or arthroscopic procedures. Although an open procedure has better visibility of the surgical field and it enables surgeons to get direct access to almost all articular lesions, open OAT has not shown superior clinical outcomes. In a cadaveric study,^6^ open and arthroscopic procedures for plug placement were comparatively conducted, and results showed no significant difference regarding accuracy and precision between the two techniques. Hence, arthroscopic OAT is the most commonly employed method.

**Fig. 1:**
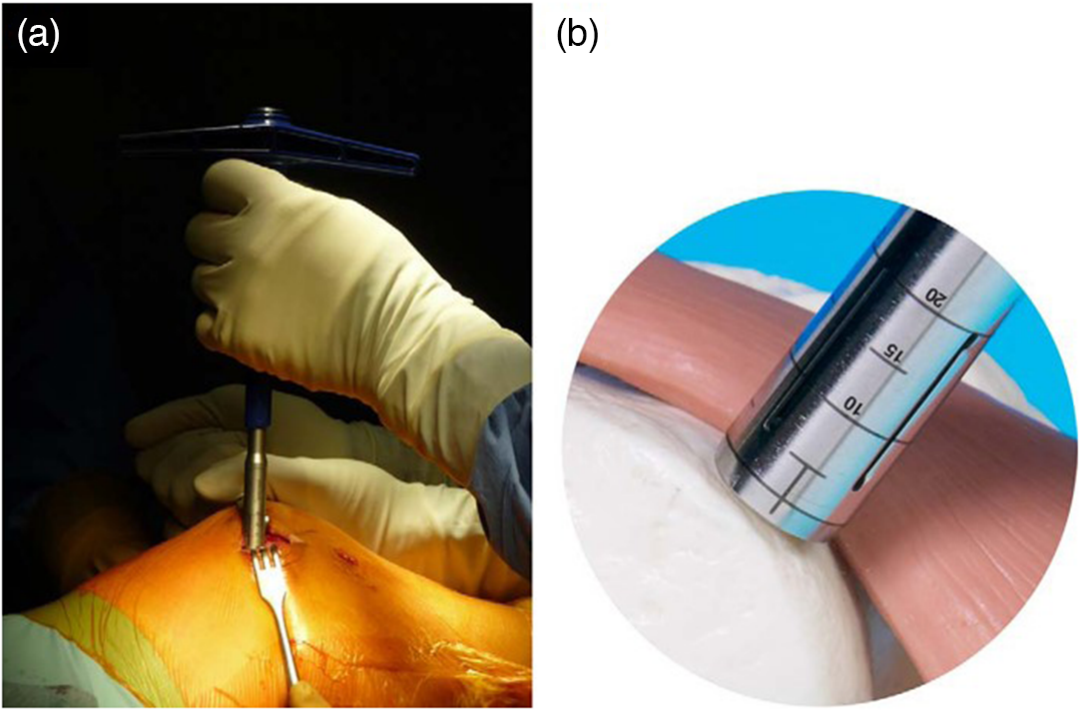
(a) Harvesting of three grafts by lateral arthrotomy from the trochlea. (b) The tubular harvesting chisel should respect the dual perpendicularity of the trochlea and remain perpendicular to the cartilage surface.^12^

In contrast to open surgery, arthroscopic OAT uses an endoscope system with a camera, a light source, and a surgical instrument that passes through a small puncture incision on the joint of the patient undergoing arthroscopic surgery. Thus, it offers considerable benefits to patients, including less post-operative pain, reduced soft tissue damage, less blood loss, shorter recovery time, and hospital stays. However, arthroscopic OAT imposes many challenges on a surgeon’s dexterity because of the well-known optical restrictions associated with a small field of view, the 30 deg optical angle of the arthroscope, and the lack of spatial awareness in a monocular arthroscope. Furthermore, numerous technical notes have reported that performing mosaicplasty arthroscopically to reduce invasiveness is an extremely demanding task. It is technically challenging to adjust cartilage thickness with three or more plugs in mosaicplasty.^5^ The congruency of the graft surface with the surrounding tissue is quite critical. If the graft surface protrudes above its surrounding, it may undergo necrosis and excessive wear,^7^ which mainly depends on the angle and depth of the graft insertion.^8^ Hence, the tubular harvesting chisel must be perpendicular to the articular cartilage surface for graft harvest and insertion to avoid differences in angulation and alignment,^9^^–^^11^ as shown in Fig. 1.

Furthermore, confusion such as hand–eye misalignment and instrument disorientation under arthroscopic guidance often occurs in surgery.^13^ Stereo vision technology can help overcome partial limitations by expanding the surgical scene with wide-angle cameras or displaying the 3D structure of the tissue. Consequently, equipping surgeons with surgical navigation assistance that eases the operational constraints of arthroscopic OAT surgery is vital. In general, position information and orientation awareness are important prerequisites for most surgical localization applications. To address this need, an ArthroNavi framework that combines electromagnetic (EM) sensing and stereo endoscopy for intraoperative instrument tracking/localization is proposed in this work.

### 1.2 Limitations of Prior Research

Many studies have made developmental efforts on instrument tracking/localization in minimally invasive procedures. Currently, the existing tracking methods mainly consist of three types: optoelectronic-based tracking, EM-based tracking, and image-based tracking. However, adopting the existing techniques for arthroscopic OAT and instrument localization remains challenging. In this section, we will discuss these popular methods and their limitations for arthroscopic OAT.

A common method for surgical localization is to use opto-electronic-based tracking systems.^14^^,^^15^ These navigation systems are currently imageless in orthopedic surgery and are generally composed of a tracker, a detector, and a computer. The pose of the instrument is calculated by the tracker and a “hand–eye” calibration matrix^16^^,^^17^ that indicates the relatvie pose relationship between the tracker and the instrument. However, these components may seem cumbersome to inexperienced surgeons. Therefore, one or more specialized technical personnel are required to support its operation. Under this circumstance, their operations have certain constraints. For example, line-of-sight between the optical tracker and markers/detector must be cautiously maintained by the surgical team to avoid optical occlusion.^18^ Nevertheless, this method is mature and is widely used in clinical procedures of orthopedics.

In contrast, an EM tracking system^19^^,^^20^ generally consists of an EM field generator placed within the surgical field, tracking sensors mounted with the surgical instruments and a monitor system. The EM field generator detects signals from the tracking sensors as the surgical instruments navigate within the surgical field and uses this signal to calculate the position and orientation of the instruments in real time. The tracking sensor is capable of tracking the position and movement of the surgical instruments without occlusion, and hence this tracking approach is free of the constraints of the line-of-sight as the EM waves can penetrate through soft tissues and obstacles within the body. However, the EM-based tracking system is prone to magnetic interference, which can influence the accuracy of the tracking data.^21^ The EM trackers rigidly mounted on the instruments must be kept away from the metallic field to make the system work without any distortion.

Compared with the aforementioned two methods, image-based tracking^22^^–^^25^ for surgical localization has been reported extensively and seems to be a low-cost and easy technique in terms of system complexity. For example, the monocular structure-from-motion or simultaneous localization and mapping (SLAM) techniques^26^^–^^28^ can be integrated with current surgical setups. These methods can estimate the 3D structure from a moving monocular endoscope and simultaneously track the pose of the endoscope. The biggest advantage of SLAM-based approaches is that no optical or EM trackers are involved. Indeed, these techniques rely on the fact that the endoscopic camera provides either sparse or dense 3D reconstructions of neighbouring tissues,^29^ which are unsuitable for arthroscopic OAT because of the following: (1) the articular surface is texture-less. An inadequate acquirement of feature points leads to a sparse point cloud that affects the accuracy of pose estimation. (2) Only the pose tracking of the endoscope is not enough for surgeons to perform OAT procedures. Registration between the endoscope, femur bone, and bone harvester must be kept consistent. (3) Another huge challenge that is unique to arthroscopic OAT is the constant extraction and insertion of the endoscope at a fast speed because of the use of various instruments, resulting in the loss of image sequences. Thus, scene initialization and mapping are required to recover the tracking during endoscope re-insertion.

### 1.3 Approach

An ArthroNavi framework that combines EM sensing and stereo endoscopy imaging for continuous and reliable instrument localization is presented in this study. Compared with the existing tracking method, the main merits of this framework are the continuous image scenes and no occlusion. Further, unlike our previous work,^30^ this framework is capable of relocation tracking navigation. This development is seen as a new technical attempt for the application of navigation in specific arthroscopic OAT surgery to improve clinical outcomes. The main contributions of this study are as follows:

1. We developed a prototype of a custom-made binocular endoscope for measuring the 3D shape of the articular surface.
2. To maximize feature matching in texture-less surfaces, a speckle module that utilizes flexi-fiber for illumination in confined spaces was developed and presented in detail. The small size enables it to be embedded in the endoscope tube.
3. A robust femur coordinate was extensively introduced for visualization and localization, which was capable of dealing with intraoperative situations such as knee joint relocation or movements.

The structure of this paper is as follows. Sec. 2 introduces a prototype of a stereo endoscope, its assistance components, and the principle of imaging and locating methods. Sec. 3 shows the experimental results associated with 3D measurement and poses localization precision. Sec. 4 presents a discussion about the current study, followed by a brief conclusion of the key significance of this study.

## 2 Methods

### 2.1 Overview

**Fig. 2:**
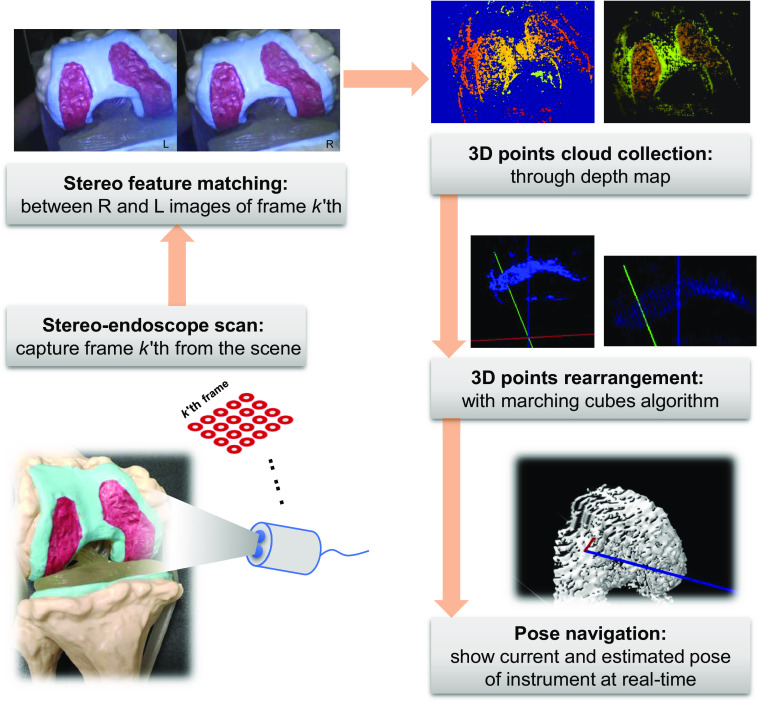
Workflow of the stereo endoscope-guided navigation framework.

In this section, the details of our proposed method are introduced thematically in terms of the workflow order. Figure 2 shows an overview of our stereo endoscope-guided intraoperative navigation framework. The proposed framework integrates EM sensing-based navigation and endoscopic vision-based navigation. It is designed with the intention of its incorporation into orthopedic workflows for operation assistance because it is a stable tracking approach that relies on a 3D points cloud obtained from a freehand stereo endoscope. Intraoperative localization of instruments can be tracked and estimated in real-time even in poor conditions that yield sparse cloud points.

### 2.2 Development of the Stereo Endoscope

Measuring a 3D shape of a knee joint in a confined space is challenging. Given that the imaging conditions and scene texture associated with arthroscopic imaging are poor, feature-matching-based passive vision (e.g., binocular vision) is not ideal. As a result, low matching accuracy makes sparse point cloud data, which is insufficient for pose estimation. Typically, active vision such as structured lighting in studies^30^^–^^32^ can overcome texture-less problems and obtain a higher matching accuracy, but some of the studies have to insert extra probes for light illumination or move the medical endoscope to achieve a 3D shape of the test object, which seems to be inappropriate for arthroscopic OAT. Consequently, we consider that passive and active methods can be combined to develop a new endoscope, which has a high measurement accuracy and does not require relative motions between the endoscope and the test object. To solve this problem, we propose applying speckle illumination in the stereo endoscope by projecting random dots onto the measured scenes to increase the accuracy of feature matching. Moreover, the illumination probe is fixed inside the endoscopic tube so the overall size of the endoscope is sufficiently small to allow it to be used in narrow spaces.

First, we adopt two customized cameras with each packaged diameter of 3.4 mm to construct a stereo endoscope based on the binocular optical model. The two cameras are mounted side by side with parallel optical axis. Taking account of the brightness inside the knee joint, the endoscope is capable of adjusting illumination by fixing four white micro-lighting emitting diodes that sit radially around the frontend of each camera. Table 1 lists the specifications of the camera used.

**Table 1:** Specifications of the customized camera.

Parameters	Values
Sensor	CMOS OV9734
Pixel size	1.4 μm×1.4 μm
Resolution	1 million pixels/720 P
Frame rate	30 frames per second
Field of view	120 deg
Depth of field	10 to 100 mm
Scan mode	Progressive

**Fig. 3:**
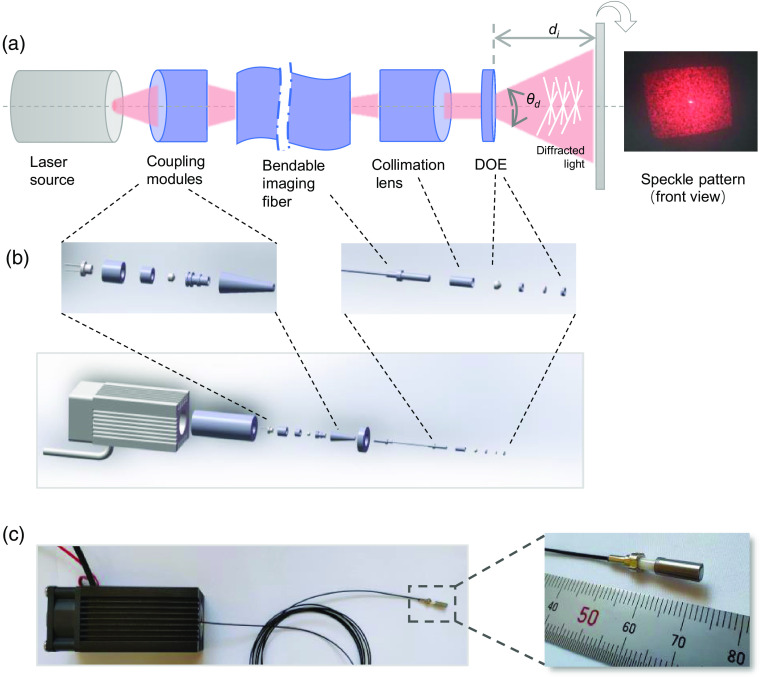
Custom-designed speckle illuminator. (a) Optical design layout of the illuminator. (b) Explosion diagram of structure composition of the illuminator. (c) Photograph of the prototype and its distal end.

Next, a custom-designed speckle illuminator is developed and presented in detail. Figure 3(a) shows the structure diagram of the custom-designed speckle illuminator. The speckle illuminator mainly consists of three components: coupling modules, collimation lens, and a diffractive optical element (DOE). The laser ray coming from the diode projector is coupled into the proximal end surface of the imaging fiber (0.22 NA, 3.5  μm core, 900  μm cladding), wherein the coupling modules consist of a TO56 and coupling lens, as shown in Fig. 3(b). On the other side of the imaging fiber, the collimation lens (F2) is used to collimate the emergent ray prior to the DOE lens. Moreover, a diffraction process used to make the ray output can be finely tuned to produce a particular speckle pattern. Figure 3(b) shows an exploded view of the speckle illuminator, and Fig. 3(c) shows the prototype of the speckle illuminator. In this study, the illuminator is originally designed to a working distance of ∼20  mm to meet the needs of arthroscopic OAT application. Based on our pre-experiments, the number of a random dot is set to ∼5000 points to generate a distinct speckle pattern for camera detection. Additional parameters about the illuminator are given in Table 2.

**Table 2:** Specifications of the custom-designed speckle illuminator.

Component	Parameter	Value
Laser source	Wavelength	650 nm
	Laser power	0 to 30 mW
	Coupling efficiency	30%
	Working voltage	12 V
	Overall dimension	95 mm×40 mm×40 mm
Bendable imaging fiber	Type	Pure silica core single-mode fiber
	Fiber diameter	3.5 μm
	Numerical aperture (NA)	0.22
	Cladding	900 μm, plastic
	Patch cord	FC/PC
	Length	1000 mm
DOE	Focal length	20 mm
	Dimension	ϕ3.5 mm×8 mm
	Number of speckle	∼5000 points
	Divergence angle	65 deg

As shown in Fig. 2, the first step in the workflow is to scan the scene and capture one proper the frame k’th by the stereo endoscope. The right and left images in frame k’th must be corrected for radial and tangential distortions before feature matching. Thus, a standard camera calibration process proposed by Zhang^33^ is conducted using the C++ platform to compute the intrinsic parameters of the two cameras. Our experiments are currently conducted without water; however, the real arthroscopic OAT surgery is performed in an aqueous environment. In that case, underwater calibration is also required as differences in the optical properties of the medium give rise to different intrinsic parameters.

**Fig. 4:**
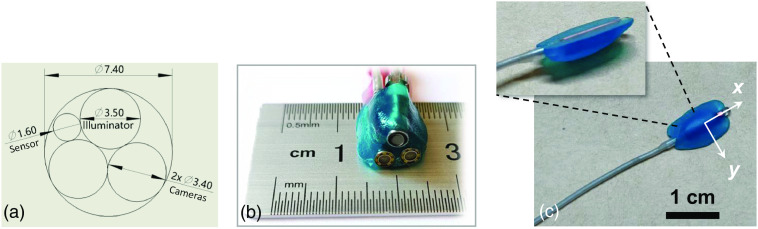
Photos of the distal end of the stereo endoscope. (a) The end-face view of the endoscopic layout. (b) A close-up shot of the endoscopic tip. (c) The correct direction of the sensor’s XOY plane.

For clarity, Fig. 4(a) shows the front view of the endoscope tip, which clearly describes the relative position of each component. Two cameras, a speckle illuminator, and an EM sensor are aligned in a circle with a diameter of 7.40 mm. The EM sensor is specified in detail in Sec. 2.4. A photograph of the endoscopic tip is shown in Fig. 4(b). Given that the sensor is a cylindrical shape, its correct position must be found before attaching it to the camera, otherwise, the relative orientation between the sensor and the camera cannot be obtained. Figure 4(c) shows the XOY plane of the sensor. Currently, the endoscope tip is mounted with light-cured resin to meet the needs of simulation experiments and to reduce the cost of using industrial-grade packaging.

### 2.3 Feature Matching

After scene capture, the next step is to establish a pixel-to-pixel relationship Rmatch:(xp,yp)l↦(xp,yp)r using epipolar lines of the two images taken by the two cameras. The semi-global matching (SGM) algorithm, which is a classical dense stereo matching approach proposed by Hirschmuller,^34^ is adopted for this process. The SGM method is based on the idea of pixel-wise matching of mutual information and approximates a global 2D smoothness constraint by combining many 1D constraints. Although numerous improved approaches have been proposed based on the original SGM algorithm, in machine vision, SGM is preferred due to its good trade-off between precision and computation requirements. Matching of images with different exposures and lighting have been tested in the original SGM algorithm. The results indicate that the average errors for matching images with differing exposures were observed to be below 20%, and those of different illuminations were ∼35%.^34^

### 2.4 Patient Tracking Coordinates and Point Cloud Collection

To obtain a successional pose estimation on the knee-joint surface, the Liberty Polhemus system (LIBERTY, Polhemus, Colchester, Vermount, United States), a state-of-the-art EM-based tracking system, is used in our ArthroNavi framework. The system consists of a transmitter and up to four sensors. The transmitter produces an EM field that acts as an accurate reference for the position and orientation measurements of the sensors.

**Fig. 5:**
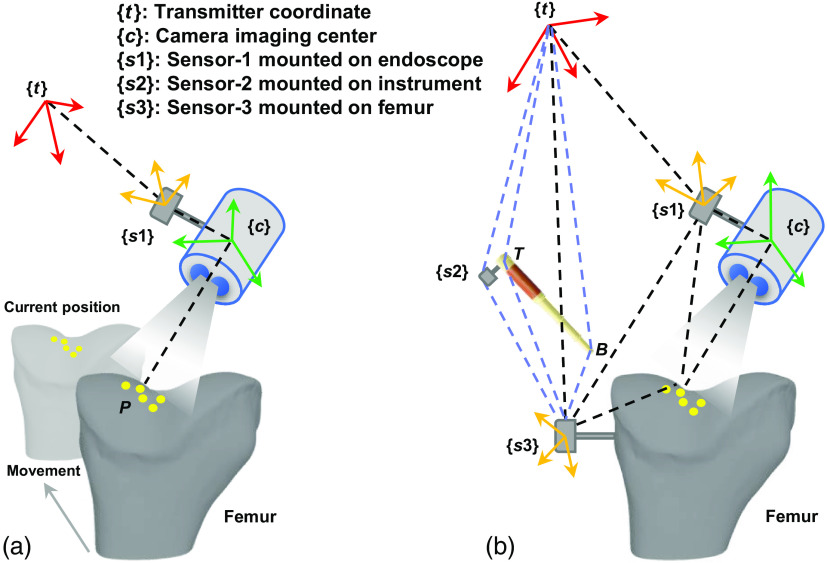
Comparison of the 3D imaging coordinate. (a) The previous tracking method. (b) Our proposed ArthroNavi tracking framework.

The patient tracking coordinate, as one of the highlights in this study, is proposed for the intra-operative robust 3D displaying. The difference between our approach and the previous imaging method utilized in another study^30^ is shown in Fig. 5, which illustrates the use of different coordinate systems. Figure 5(a) shows the previous imaging method. When the femur surface is scanned and calculated by the endoscope, the surface will be incrementally displayed in the transmitter coordinate system. Let $P_{i}\in R^{3}$, i∈{1,2...,n} denote a point set in the minimally invasive surgery (MIS) scene, and Pci be the corresponding point cloud produced from the endoscopic camera system. The 3D point cloud can be transformed to the transmitter’s view by the following equation:

Pt=Rs1t(ds1+Rcs1·Pc)+Pts1, (1)

where Rs1t and Pts1 are the relative orientation and position of sensor-1 with respect to the transmitter coordinate system; both of them can be obtained from the Liberty EM tracking system. Rcs1 denotes the relative orientation of the camera with respect to the sensor-1 coordinate, and ds1 is the relative distance between the sensor-1 and the camera.

Although the scanned surface can be displayed based on the transmitter system, some inevitable situations may throughout the surgical operation. For example, (1) slight backward or forward movement of the femur during orthopedics operation and (2) orthopedists may knock against the transmitter. As a result, the position and orientation of the obtained 3D surface do not match that of the femur in the current position, as shown by the yellow points in Fig. 5(a). Hence, subsequent localization would not have succeeded on the femur surface.

For our proposed framework, as can be seen in Fig. 5(b), we add an additional sensor-3 to the femur. In particular, sensor-3 is mounted rigidly to minimize the influence of specimen motion. By doing so, sensor-3 can be considered as the femur coordinate that takes the role of patient tracking. Thus, in contrast with Fig. 5(a), the transmitter here will change to an intermediary, which allows us to achieve great robustness. A 3D point in the MIS scene P can be transformed into the femur coordinate as follows:

Ps3=Rts3(Rs1t(ds1+Rcs1·Pc)+Pts1)+Ps3t, (2)

where Rts3 and Ps3t are the relative orientation and position of the transmitter with respect to sensor-3. Given that Rts3=Rs3t−1=Rs3tT and Ps3t=−Rtts3Ps3=−Rs3tTtPs3 are known, **Rts3 and Ps3t can be calculated. By utilizing the local patient tracking coordinate as an agent, we solved three vital issues for intraoperative localization in arthroscopic OAT: (1) intraoperative femur can be rotated or moved anywhere at the correct pose, which provides the orthopedist with operation conveniences, (2) any collision with the transmitter does not affect the localization results, and (3) no pre-operative registration or initialization is required, which saves time.

### 2.5 3D Points Rearrangement

We rearrange the point cloud to reconstruct surfaces locally using the refined marching cubes algorithm.^35^ Unlike the classical marching cubes algorithm,^36^ this method is a non-interpolation approach, which decreases the computational cost. Let a point set Ps3i∈R3, i∈{1,2,…,n} be the point cloud represented under sensor-3 coordinate. The smooth and continuous surface S can be reconstructed by a two-step procedure: (1) a series of interval-planes are defined as follows:

$$I_{i}=\{x,y\in R^{3}|Ax+By-D_{i}(t)=0\},$$ (3)

where $D_{i}(t)=kt+\min\;z_{i}$, k∈{0,1,…,n}, and $\forall \;z_{i},D_{i}(t)\in [\min\;z_{i},\max\;z_{i}]$, and t>0 is the interval parameter of planes. Meanwhile, t is also the edge interval in the plane. Using Eq. (3), a cubic grid is created among the point cloud. (2) For each point located in a cube, projections along three directions will be calculated. A point will be rearranged to the adjacent vertex if the projection distance is less than half of the interval on each axis. Otherwise, the point will be rearranged to the next vertex. Accordingly, based on an eight-bit indicator (which is equal to one with a point and zero without points), we can extract the local isosurface and then reconstruct the entire surface.

### 2.6 Pose Computation

After surface recovery, a geometrical constraint method that utilizes the surface 3D points is used to compute a pose for the intraoperative instrument. In the case of arthroscopic OAT surgery, the orientation of the instrument is more important than its position because the position information can be obtained by the tracking system. The normal vector of every position on the reconstructed surface represents the best insertion or extraction orientation of the current instrument. Based on the proposed tracking framework, shown in Fig. 5(b), the current pose of the instrument can be shown by the vector $\overset{\to }{TB}$ using points T and B, which are expressed as follows:

Ps3T=Rts3(Rs2t·Ps2T+Pts2)+Ps3t, (4)

and

Ps3B=Rts3(Rs2t·(Ps2T+TB¯)+Pts2)+Pts3, (5)

where PTs2 and $\overset{¯}{TB}$ can be measured beforehand.

As introduced in Sec. 2.4, 3D points $P_{i}$ in the MIS scene are matched and transformed to the femur coordinate sensor-3, which is discrete character data. Thus, we approach the normal vector problem through a cross-product that computes two arbitrary vectors, i and j, built by a triangle. Consequently, the problem becomes that of finding an inscribed triangle Δs whose cross product is the best approximation of the normal vector $n_{e}$ on current position

$$\underset{\Delta s}{\min}\|k-n_{e}\|,$$ (6)

where k=i^j. Notably, the robustness of the triangle finding is increased with a large area. However, when the triangle area Δs→0, the confidence level of approximation will be higher. Therefore, the parameter of the searching area in the point cloud is defined as ∼0.6  mm in this study. Based on this geometrical constraint, the pose and normal vector can be simultaneously obtained and shown according to different positions of the 3D surface. However, even if these two vectors are overlapped, the discrepancy between measurements and theory remains because the real normal vector is unknown. Therefore, the procedure of pose evaluation is required, as shown in the following section.

### 2.7 Pose Assessment Method

**Fig. 6:**
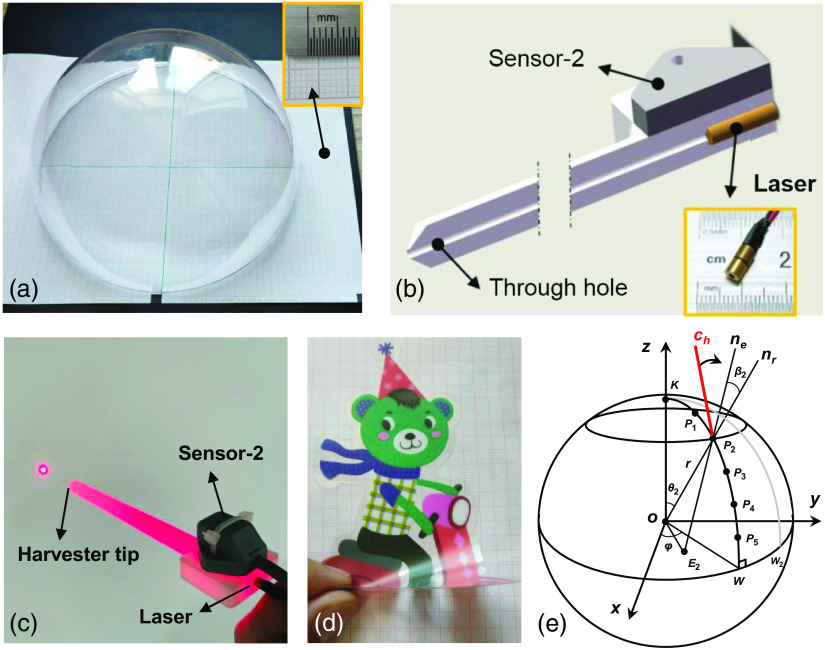
Precision evaluation method for pose estimation. (a) Photograph of the hemispherical shell with an ECG paper. (b) Cutaway view of the custom-designed component harvester. (c) A laser point, passing through the hole of harvester, is projected onto a white paper. (d) A texture sticker is affixed to the hemispherical shell using static electricity. (e) The computational principle of the pose errors.

To evaluate the accuracy of pose estimation, we proposed an ingenious hemisphere tabulation method for pose measurement. Figure 6 shows the principle of the evaluation method. A standard hemispherical shell with a radius of 98.0 mm and a recording paper of electrocardiogram (ECG) is used for the pose test and calculation, as shown in Fig. 6(a). Since the femur profile is a free-form surface and autografts are generally harvested from a smooth area,^4^^,^^5^ a similar size of hemispherical shell is selected to match the real-life femur dimensions. In particular, a highlight of this method is that the hemispherical shell is designed to be transparent, which makes the instrument pose recorded through an optic projection technique. To this end, a specific component that imitates a bone harvester in the OAT surgery is designed using an acrylic board. The 3D structural drawing is shown in Fig. 6(b). A point laser module (dimension: 3.8  mm×13.8  mm, 1 mW, 650 nm) is embedded at the top end of the component. The axis of laser light must be arranged co-axially with that of the component. Besides, an EM sensor-2 assisted in pose navigation is mounted close to the top end of the component, as shown in Fig. 6(c). Figure 6(d) gives a close-up view of the texture sticker. Prior to frame capture, this sticker is affixed to the hemispherical shell using the electrostatic adsorption to capture as much of feature matching as possible.

Figure 6(e) shows the definition of the spherical coordinate system. The XOY plane is located on the ECG paper, which is fixed throughout the experiment. An arc $\overset{⌢}{KW}$ passing the sphere center is evenly divided into six sections by five points, $P_{1},P_{2}\ldots ,P_{5}$, which are marked on the shell in advance. φ is a horizontal rotation angle with respect to x direction and is defined as π/12 during the experiment. Increasing the number of arc with an interval of angle φ, for example $\overset{⌢}{KW_{2}}$, the sample size becomes bigger, which brings higher evaluation efficiency. Geometrically, given that the central angle of a circle is the same degree as the opposite arc, we obtain the following

$$\theta_{s}=\frac{\overset{⌢}{KP_{s}}}{r}=\frac{s\pi }{12},$$ (7)

where s∈{1,2...,5} is a section index on the arc and r is the spherical radius. Based on the spherical coordinate system, the 3D coordinates of the five marker point $P_{s}$ on one arc can be expressed as follows:

$$[\begin{array}{c} x_{P_{s}}\\ y_{P_{s}}\\ z_{P_{s}} \end{array}]=[\begin{array}{c} r\;\sin\;\theta_{s}\;\cos\;\varphi \\ r\;\sin\;\theta_{s}\;\sin\;\varphi \\ r\;\cos\;\theta_{s} \end{array}].$$ (8)

For each measurement of the positions on the arc, data acquisition yield a set of data samples. The pose accuracy is determined by computing these measurements.

In practice, once the frame capture is done, the texture sticker will be removed from the shell surface. When the havester tip comes in contact with the shell surface, the navigation process is triggered, as shown in Fig. 6(e). At this stage, the current pose of the harvester $c_{h}$ and the estimated normal vector $n_{e}$ are displayed on the monitor during the navigation process. When these two vectors coincide, the pose represents the best fit for the current position. Meanwhile, a ray line representing the component pose is projected point to $E_{2}$ onto the ECG paper and the corresponding coordinate is recorded. Thus, based on the law of cosines, the error angle $\beta_{2}$ between the estimated $n_{e}$ and its real $n_{r}$ can be computed using the following equation:

$$\beta_{2}=\arccos(\frac{{\overset{¯}{E_{2}P_{2}}}^{2}+r^{2}-{\overset{¯}{OE_{2}}}^{2}}{2r\overset{¯}{E_{2}P_{2}}}),$$ (9)

where $\overset{¯}{VT}$ denotes the Euclidean distance of 3D points V and T.

## 3 Experiments and Results

We designed a two-part quantitative and qualitative evaluation process: (1) using a series of standard objects of flat-plane, surface, and sphere to evaluate the performance of the stereo endoscopic reconstruction error and the accuracy of the proposed ArthroNavi framework and (2) using a full-size femur model (SawBones.org) to assess the feasibility of our proposed framework.

### 3.1 Implementation Settings

The measurement system was implemented in a Windows 10 20H2 environment using C++ [without any graphics processing unit (GPU) acceleration] by three projects. All experiments were conducted on a laptop equipped with Intel Core 2.7 GHz CPU, 8 GB Memory, and 1 Intel HD 620 graphics card. To accelerate data reading between the three projects, a shared memory technique was adopted for inter-process communication. SGM with our proposed framework runs in real-time at 200 frames per second on average, and the 3D surface render process takes ∼900  μs.

### 3.2 Precision Analysis of the Endoscope

**Fig. 7:**
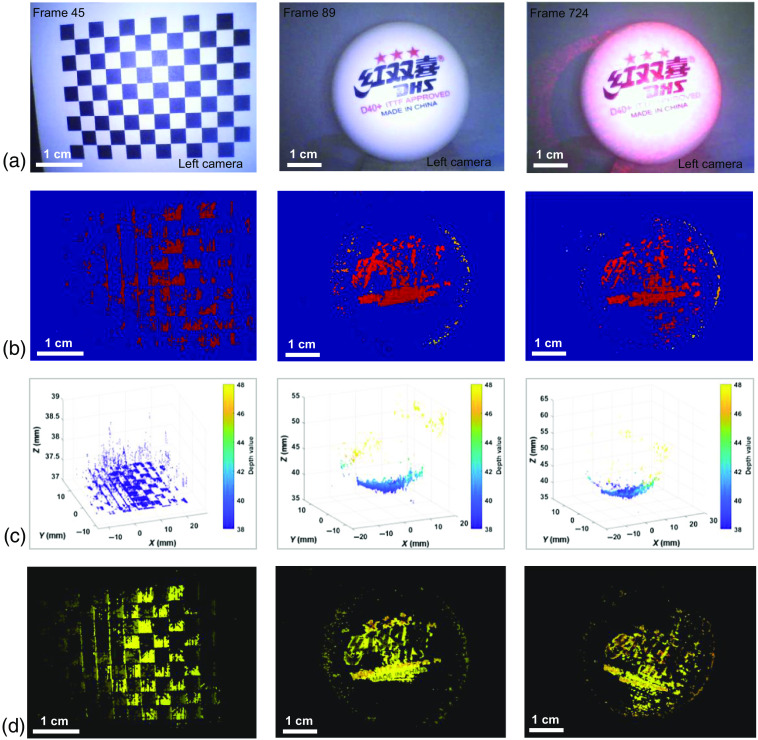
3D measurement results of a chessboard flat and a ping-pong. (a) The photographs of tested objects. (b) The corresponding depth maps of the tested objects. (c) The representations of the depth maps. (d) The corresponding texture mapping of the point cloud.

First, to evaluate quantitatively the reconstruction accuracy of our self-development endoscope, a chessboard flat with a pattern size of 36×27  mm and the highest quality (3-start) ping-pong with a diameter of 40.09 mm were measured. The endoscope was fixed on a mount, and the measuring distance was ∼3.7  cm for the chessboard and 4.0 cm for ping-pong. Figure 7(a) shows the corresponding objects captured by the stereo endoscope. For a clear observation, only the captured images by the left camera were shown. Figure 7(b) shows the corresponding depth maps, and Fig. 7(c) shows the 3D plot of the depth maps. Furthermore, based on the depth maps, the point cloud mapping with texture was obtained, as shown in Fig. 7(d). The depth maps obtained from the C++ project were initially in 2D format. To visualize them in 3D and enable features such as shape rotation and zooming, we utilized a third-party software. This allowed us to display the texture-mapped images in a more comprehensive manner. By doing so, we were able to select an optimal point cloud for use in the subsequent navigation process.

Besides, for comparison results, 3D imaging with a speckle pattern on the ping-pong ball was conducted, as shown in Fig. 7. The measurement parameters were the same as those without speckle patterns. Figure 7(b) shows that the edge of the ball with pattern illuminated is obviously sharper, and the obtained feature points with no texture on the ball become richer. Therefore, using a speckle illuminator, the endoscope was prone to obtain a relatively big area 3D shape.

**Fig. 8:**
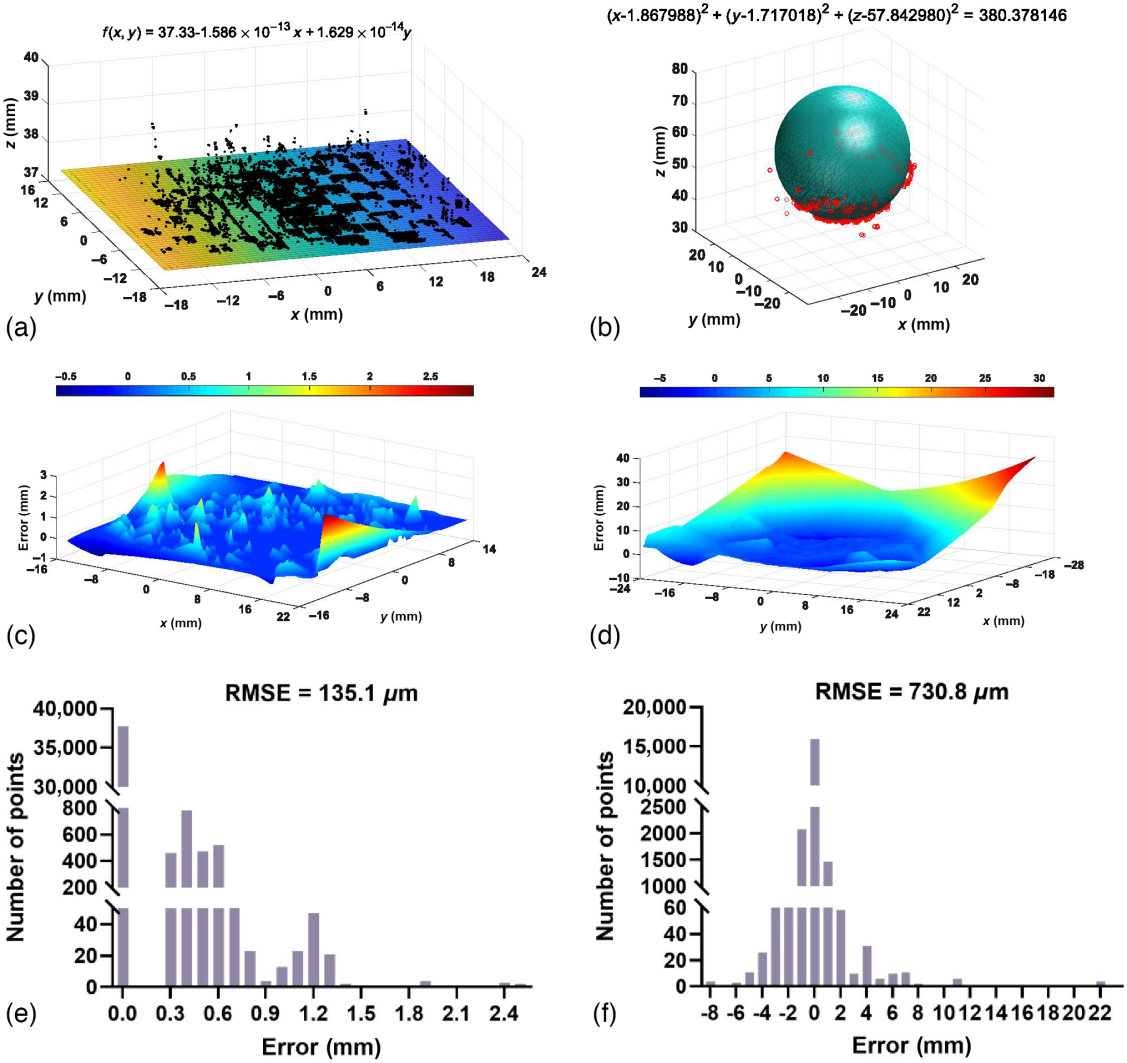
Precision analysis for measuring a chessboard flat and a ping-pong. (a) The fitting results of a chessboard flat. (b) The fitting results of a ping-pong. (c), (d) The corresponding distribution of the measured errors of (a) and (b). (e), (f) The corresponding quantitative histograms of the measured errors of (a) and (b).

3D measurement units are commonly evaluated by a set of artifacts with a common geometry, such as planes,^37^ spheres,^38^ and cones.^39^ Even a liquid crystal display was assumed as a flat plane specimen to evaluate the precision of a compact 3D measurement unit.^40^ Similar to references,^26^^,^^41^^,^^42^ the method of fitting and statistic was adopted in the present study for reconstruction precision analysis. Based on the acquired 3D point cloud, the plane fitting by a polynomial was conducted to obtain the ideal plane ($R^{2}=0.9998$) as the ground truth, as shown in Fig. 8(a). The difference between the ideal plane and the measured plane was calculated to obtain the 3D measured errors. The two surfaces were aligned in the endoscope coordinate system and we get a series of (x,y) coordinate points based upon the surface area, and then compare the distance of the depth value, z, of the two surfaces. By doing so, we can obtain a point-by-point 3D measured error. Figure 8(c) shows the measurement error of the chessboard plane. Besides, the quantitative histograms of the differences were shown in Fig. 8(e). The statistical results showed that the major measurement errors were <1.3  mm with the root mean square error (RMSE) of 135.1  μm. Similarly, for the 3D measurement of the ping-pong, the sphere fitting [Fig. 8(b)] was adopted to obtain the actual measurement errors, as shown in Fig. 8(d). The fitting diameter of the 3D point cloud was 39.01 mm, which was 1.08 mm deference compared with that of the ping-pong. Moreover, the RMSE of the 3D measurement accuracy was ∼730.8  μm, as shown in Fig. 8(f).

A comparison of reconstruction accuracy for different 3D endoscopic systems was summarized in Table 3 in ascending order of the published year. The considered aspects are the imaging technique, system setup complexity, mean error, and working distance. Note that these selected endoscopic measurement systems, the distal end, are small in size and developed for applications in MIS. Although having a small distal end, for those endoscopic measurement systems that are fixed on a desktop measuring platform^47^^–^^49^ are excluded for comparison. As seen in Table 3, notably, most of these endoscopic systems have a sub-millimeter accuracy in 3D reconstruction. A relative large working distance results in a smaller max error. Consequently, for handheld endoscope systems, the working distance is a main aspect that affects the imaging accuracy and decides the potential use in clinical applications. Our previous study^30^ is more accurate than current method because it employed a monocular system structure. On the other hand, such a co-axial system is susceptible to vibration interference, which can lead to structured light (SL) projection becoming out of focus.

**Table 3:** Precision results comparison for different 3D endoscope measurement systems.

Year	Methods	Imaging technique	System complexity	Mean error (mm)	Max error (mm)	Working distance (mm)
2006	Hayashibe et al.^38^	Mono + SL	Medium	0.16	1.92	150 to 160
2014	Kumar et al.^43^	Mono + 3D CT model	Low	1.08	1.78	Not given
2015	Edgcumbe et al.^37^	Mono + SL	Low	1.40	2.50	166 ± 7
2015	Yang et al.^44^	Mono + 3D US^a^ image	High	0.11	0.19	∼180
2015	Lin et al.^45^	Mono + SL	High	0.67	5.04	Not given
2018	Chen et al.^26^	Mono + SLAM	Low	2.54	Not given	Not given
2018	Lin et al.^46^	Mono + SL	High	0.64	3.19	15 to 40
2020	Sui et al.^42^	Stereo + SL	High	0.13	0.18	Not given
2021	Long et al.^30^	Mono + SL	Low	0.15	0.24	2 to 21
2023	Proposed method	Stereo + SL	Medium	0.14	4.00	37 to 40

a US denotes the ultrasound.

SL: Structured Light; CT: Computed Tomography.

### 3.3 Precision Analysis of the Pose

After the shell surface was captured and reconstructed, pose evaluation with a freehand component was conducted. First, the endoscope was fixed at a specific position with an imaging distance of ∼4.0  cm. Imaging a transparent shell with a stereo endoscope was slightly challenging. Thus, a small texture sticker with pattern was on the shell surface for scanning to maximize feature matching. Subsequently, pose navigation was carried out on the shell surface randomly.

**Fig. 9:**
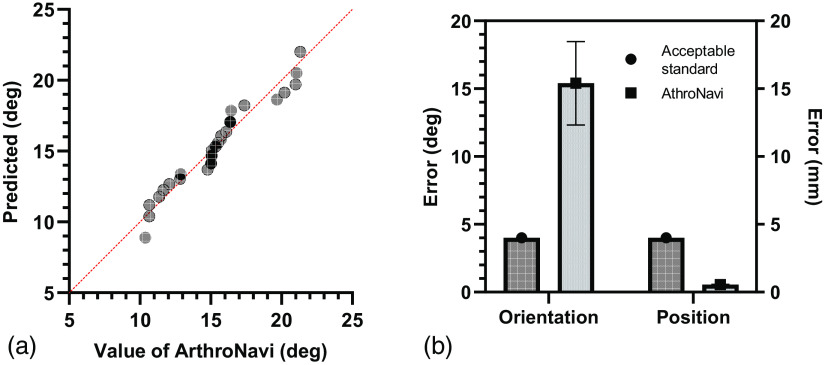
Comparison results on instrument pose evaluation. (a) Normal distribution of the pose evaluation test on a hemispherical shell. (b) Navigation disparity between the ArthroNavi and the clinically acceptable standard.

Experimental results suggested that the mean errors of pose localization were 15.4 deg (range of 10.3 deg to 21.3 deg), with a standard deviation (SD) of 3.08 deg. Figure 9(a) shows the normal distribution of the pose estimation results. This analysis was based on 30 position estimations of 6 different static endoscope captures. For each capture, five estimations on different positions (i.e., interval angle φ) were computed. In contrast, the errors observed in a freehand technique performed by experienced surgeons during an arthroscopic surgery study^50^ comparing computer-assisted navigation to the freehand technique were measured at 14.8 deg (range of 6 deg to 26 deg), with a SD of 7.53 deg. These results were derived from a hypothesis that the computer-navigated method would offer greater precision in positioning with respect to the perpendicularity of the grafts relative to the joint surface when compared to the freehand arthroscopic technique. After graft transplantation was performed using the freehand approach, positioning accuracy was assessed in a similar fashion to the navigated procedure, facilitating a direct comparison. With regard to the experimental results, our proposed method was able to achieve results within the same order of magnitude as those accomplished by experienced surgeons. Besides, instrument pose conducted by the freehand technique mainly relied on the dexterity and expertise of the surgeon, which may yield nonuniform results, whereas those in our method were automatic and identical. Nonetheless, according to the quantitative score table for guide concepts proposed by Audenaert et al.,^51^ the pose results obtained in our method and the freehand technique are both beyond the clinically “acceptable” range (i.e., error <4  deg and 4 mm). Therefore, achieving greater accuracy will be necessary. Figure 9(b) shows the navigation disparity between our ArthroNavi and the clinically acceptable standard.

Table 4 summarizes a comparison of pose accuracy for different instrument localization methods in descending order of the orientation accuracy. Numerous studies on pose estimation have been reported; these studies were selected for comparison because they fell in the field of bone surgery, especially in orthopedics. It should be noted that the position error in our proposed method is cited from the study of Polhemus EM tacking calibration.^57^

**Table 4:** Pose accuracy comparison for different instrument localization methods. The results are given as mean ± SD.

Year	Methods	Orientation error (deg)	Position error (mm)	Specific application
2021	Hu et al.^52^	1.07 ± 0.25	4.94 ± 0.23	Knee joint surface tracking
2018	Gadwe et al.^53^	1.50 ± 0.87	1.29 ± 0.67	Pose estimation of endoscope
2021	Hu et al.^54^	2.13 ± 0.81	3.64 ± 1.49	Typical knee drilling tasks
2020	Chen et al.^55^	2.55 ± 0.49	2.54 ± 0.15	Robot-assisted spine surgery
2020	Kügler et al.^56^	6.59 ± 10.36	0.75 ± 0.82	Pose estimation of a screw
2012	Benedetto et al.^50^	14.8 ± 7.53	Not given	Grafts harvest/placement
2023	Proposed method	15.4 ± 3.08	0.55 ± 0.02	Grafts harvest/placement

### 3.4 Phantom-Based Validation

**Fig. 10:**
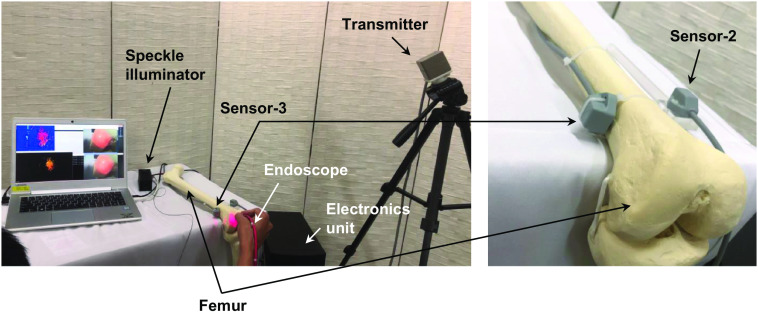
Experiment setup for femur model imaging.

A femur model (normal size) was used for the validation of the stereo endoscope-guided navigation framework. Figure 10 shows the experimental configuration on the femur equipped with the proposed tracking method. The endoscope tip position for scene capture during the test was ∼3  cm from the femur surface. Unlike the previous ping-pong ball test, the femur surface was captured with a freehand endoscope, which was in accordance with conditions used in medical applications. Please note that the hypothesis of phantom measurement was that the navigated normal vector $n_{e}$ obtained from Eq. (6) would be considered the gold standard for assessing the perpendicularity of the current position of the joint surface (i.e., $n_{r}≐n_{e}$).

**Fig. 11:**
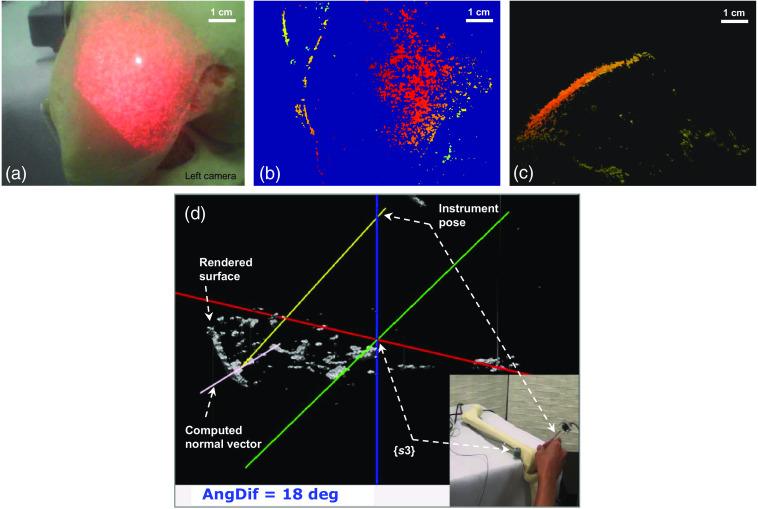
Experimental results on a femur model. (a) A capture of the femur surface with a speckle pattern. (b) The corresponding depth map. (c) Side view of the 3D point cloud. (d) Snapshot from a real-time navigation video of the femur model. The instrument pose (yellow line) was synchronized with that instrument holed in hand (Video 1, MP4, 7.78 MB [URL: https://doi.org/10.1117/1.JBO.28.10.106002.s1]).

Based on our framework procedure, only one frame was captured for 3D imaging, as shown in Fig. 11(a). Owing to the speckle pattern illumination, the femur surface was imaged successfully in a few seconds. The corresponding depth map and a side view of the 3D point cloud were shown in Figs. 11(b) and 11(c). Once the frame was captured and saved, the endoscope task has been completed. The scene capture was a static scan without any trajectory movement. The point cloud data were loaded into another C++ project that uses OpenGL for 3D surface rendering. Figure 11(d) shows a snapshot of the reconstructed surface and the instrument localization. The surface and the instrument pose were displayed based on the sensor-3 coordinate system, which was able to anti-shifting positioning. The number displayed in AngDif represents the angle between the instrument’s current pose $c_{h}$ and the estimated normal vector $n_{e}$ used for pose adjustment. Furthermore, the graphical user interface was capable of shape rotation and zooming ability. A real-time navigation video was provided by authors in Video 1. Given the showcasing based on the femur coordinate that was introduced in Sec. 2.4, our proposed method was capable of movement tracking navigation.

## 4 Discussion

The current work introduces a method to create a stereo image with a self-developed endoscope and generate an image-based pose localization, with the intention for this system to be used for arthroscopic OAT surgery. The current framework can work with 30 frames per second on a laptop without a supporting GPU.

Various algorithms are available for feature matching, and we utilized the classical one proposed by Hirschmuller, which is more accurate and faster than other improved algorithms. In recent years, a number of 3D reconstruction algorithms^58^^,^^59^ based on convolutional neural network (CNN) have been presented. These methods are capable of real-time camera tracking and dense mapping after the model is trained by a huge database of labeled images. An ideal scenario is that such techniques could be integrated into the endoscope and correlate with the positions of arthroscopic surgical instruments. However, the feature in the knee joint scene is inherently poor, and model training based on a huge image database requires manual labeling of the images, which is impractical and time-consuming. Hence, the CNN-based tracking method was not our choice for image mapping.

EM-based tracking enable localization of pre-operative instruments within a patient’s body without line of sight. The EM tacking accuracy and robustness is a challenge in the clinical application. However, a recent study that applied the well-established standardized assessment protocol^60^ to the Polhemus EM tracker demonstrated that the mean orientation error was found to be 0.1 deg in a laboratory environment, and the distance accuracy stayed in the sub-millimeter range on an average of 0.55±0.02  mm. Precision and orientation accuracy do not seem to be affected by instrument tracking.^57^ The source of error in instrument locating stemmed from the error in endoscope images. No cumulative error was yielded during the entire navigation process. Furthermore, the pose estimation method that searched three 3D points in a ∼0.6  mm diameter circle was considered precise. This search method even works in the current sparse 3D point cloud. Thus, improving the quality of the 3D reconstructed surface would give a better localization result. To this end, we have several choices: (1) using a computational mask to filtrate image noises or abnormal points and (2) replacing the customized cameras with commercially available apparatus because customized cameras lack stability and synchronization.

While the proposed framework was developed based on the consideration of clinical requirements of arthroscopic procedures, it could be used for some general applications in diagnostic and endoscopic interventions. For example, the framework could be applied to instrument alignment and bone harvesting in femoral head replacement or entire hip replacement.

### 4.1 Limitations and Future Works

The main limitations of the proposed method include the following: (1) although the custom-made monocular camera has a good imaging quality at a distance of 1 cm, the optimal imaging distance of the stereo endoscope (4 cm) is beyond that of arthroscopic practice application. (2) Only a part of the femur shape was imaged and navigated for surgeons, which may lead to direction absence. (3) In the current framework, although the localization error of 15.4 deg was the same level as that of a freehand technique (14.8 deg), the localization accuracy is not so satisfied because of the sparse 3D point cloud that came from the noise in endoscope images.

Stereoscopic-image-based navigation is challenging and achievable and will be investigated in future work. One possible solution to improve imaging accuracy is to adopt higher-definition cameras and use a shared input port to solve the frame alignment problem and yield more accurate feature matching. The point number of 5000 for the current speckle module remains excessive in consideration of the ideal imaging distance of an endoscope in practice application. Based on our experience, ∼2500 points are suitable for the ideal imaging distance of 2 to 3 cm, which enables cameras to detect an unambiguous pattern. Alternatively, the colored checkerboard pattern^37^ or randomly distributed spots with different colors^46^ are potential for better imaging.

## 5 Conclusion

This study contributes to existing clinical needs by developing a practical instrument localization approach that is non-disruptive to the operation process. Particularly, it proposes a complete framework for intraoperative navigation by combining reconstructed 3D surface and external trackers to bridge the gap in the application of existing tracking methods to OAT surgery. The pose localization method was validated by standard models and phantom femur. The 3D surface reconstruction is promising, and the pose navigation is operated in real-time. We hope that this prototypical framework can enlighten a new computer-aided direction for the treatment of cartilage damage in the knees.

## Data, Materials, and Code Availability

All relevant data, materials, and software code used in this research are available upon request from the corresponding authors.
